# Therapeutic Lessons to be Learned From the Role of Complement Regulators as Double-Edged Sword in Health and Disease

**DOI:** 10.3389/fimmu.2020.578069

**Published:** 2020-12-10

**Authors:** Esther C. W. de Boer, Anouk G. van Mourik, Ilse Jongerius

**Affiliations:** ^1^Sanquin Research, Department of Immunopathology, and Landsteiner Laboratory, Amsterdam University Medical Centre, Amsterdam Infection and Immunity Institute, Amsterdam, Netherlands; ^2^Department of Pediatric Immunology, Rheumatology, and Infectious Diseases, Emma Children’s Hospital, Amsterdam University Medical Centre, Amsterdam, Netherlands

**Keywords:** complement, complement regulators, complement therapeutics, complement antibodies, complement-mediated disease

## Abstract

The complement system is an important part of the innate immune system, providing a strong defense against pathogens and removing apoptotic cells and immune complexes. Due to its strength, it is important that healthy human cells are protected against damage induced by the complement system. To be protected from complement, each cell type relies on a specific combination of both soluble and membrane-bound regulators. Their importance is indicated by the amount of pathologies associated with abnormalities in these complement regulators. Here, we will discuss the current knowledge on complement regulatory protein polymorphisms and expression levels together with their link to disease. These diseases often result in red blood cell destruction or occur in the eye, kidney or brain, which are tissues known for aberrant complement activity or regulation. In addition, complement regulators have also been associated with different types of cancer, although their mechanisms here have not been elucidated yet. In most of these pathologies, treatments are limited and do not prevent the complement system from attacking host cells, but rather fight the consequences of the complement-mediated damage, using for example blood transfusions in anemic patients. Currently only few drugs targeting the complement system are used in the clinic. With further demand for therapeutics rising linked to the wide range of complement-mediated disease we should broaden our horizon towards treatments that can actually protect the host cells against complement. Here, we will discuss the latest insights on how complement regulators can benefit therapeutics. Such therapeutics are currently being developed extensively, and can be categorized into full-length complement regulators, engineered complement system regulators and antibodies targeting complement regulators. In conclusion, this review provides an overview of the complement regulatory proteins and their links to disease, together with their potential in the development of novel therapeutics.

## Introduction

### The Complement System

Upon its discovery at the end of the 19^th^ century, the complement system was considered to consist of only one component. Nowadays it is known that the complement system is a complex part of the innate immune system, consisting of a large number of proteins and associated regulators (1, 2). In order to respond quickly to pathogens, the components of the complement system are present in plasma and thus are readily available throughout the body (3, 4). Three different pathways can initiate complement activity: the classical pathway (CP), the lectin pathway (LP) and the alternative pathway (AP). Activation of these pathways takes place *via* antibody-binding, recognition of specific sugar patterns or spontaneous C3 hydrolysis, all resulting in formation of a C3 convertase. C3 convertases cleave C3, resulting in opsonization of pathogens and formation of C5 convertases. With C5 convertases the terminal pathway is initiated which will result in chemotaxis and formation of the membrane attack complex (MAC) (3).

**Figure 1:**
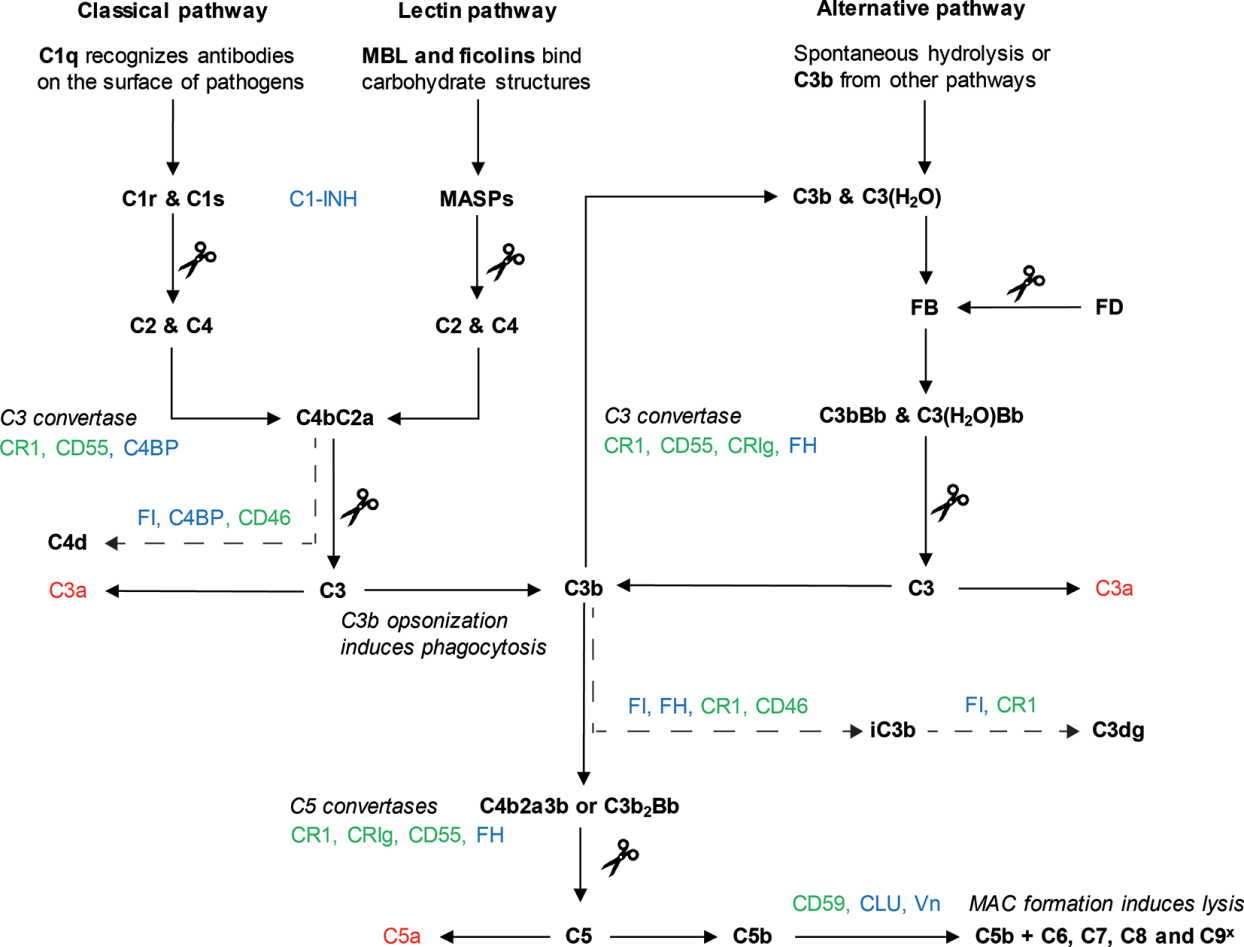
The complement system. Three pathways can lead to complement activation: the classical pathway (CP), the lectin pathway (LP) and the alternative pathway (AP). Activation of the CP (left) starts with binding of C1q to target cells, often *via* antibodies. Binding of C1q leads to cleavage of C1r, which in turn cleaves C1s. The proteolytic activity of C1s results in the cleavage of C2 and C4. These components form the C3 convertase of the CP, C4bC2a. Activation of the LP (middle) starts with the binding of MBL or ficolins to carbohydrate structures on target cell surfaces. As a result, the proteases of the LP, MASPs, cleave C2 and C4, also leading to the formation of C4bC2a. The AP pathway (right) is activated by spontaneous hydrolysis of C3, which leads to the formation of C3(H_2_O), or by C3b from the CP or LP. After assembly of C3b, FB binds to C3b or C3(H_2_O) and is cleaved by protease FD. This results in the formation of the C3 convertases of the AP, C3bBb or C3(H_2_O)Bb. C3 convertases of the CP/LP and the AP cleave C3 into C3b and C3a. Usage of C3b in the AP causes an amplification loop, which can amplify the CP and LP. In addition, C3b deposition leads to formation of two C5 convertases: C4b2aC3b (CP/LP) or C3b_2_Bb (AP). C5 convertases cleave C5 in C5a and C5b. C5b is used for the formation of the MAC which is formed after assembly of C6, C7, C8 and multiple C9 molecules (C9^x^). The MAC is inserted into the membrane to induce lysis. C3a and C5a (depicted in red) are anaphylatoxins important for chemotaxis and the initiation of inflammation. Complement components are depicted in bold, scissors indicate cleavage. Complement regulators can be divided in two groups: soluble (depicted in blue) and membrane-bound (depicted in green). These regulators prevent complement activation by targeting the complement pathway in different places, as depicted here. The inactivating breakdown pathways of C3b and C4b respectively by complement regulators are indicated here with a dotted arrow, showing the different breakdown products.

Although all three complement pathways result in the formation of a C3 convertase, their initiation and intermediate steps differ (**Figure 1**). The CP is mainly initiated by antibody binding to target cells. The C1 complex consists of C1q, C1r and C1s. C1q is the pattern recognition molecule, and upon surface binding of C1q, the protease C1r is activated, cleaving and activating C1s. C1s can then cleave C2 and C4, which leads to the formation of C3 convertase C4bC2a (3, 5, 6). The LP is activated in a similar way, with ficolin and mannose-binding lectin (MBL) acting as pattern recognition molecules. These molecules recognize microbial carbohydrate structures. Upon recognition, the MBL-associated serine proteases (MASPs), can cleave C2 and C4, to form the C4bC2a C3 convertase (5–7). Lastly, the AP is activated by C3b coming from the other two pathways. In addition, constant background spontaneous hydrolysis of C3 results in formation of C3(H_2_O) which also serves as a platform for the AP. C3b or C3(H_2_O) will bind Factor B (FB), which is cleaved by the protease Factor D (FD), leading to the formation of C3bBb, another C3 convertase (5, 8). The AP mechanisms enable it to work as an amplification loop for the CP and LP (9, 10).

After the formation of a C3 convertase, the three pathways all continue with the terminal complement pathway. The C3 convertases cleave C3 into C3a and C3b, which leads to the formation of two different C5 convertases: C4b2a3b and C3b_2_Bb. The C5 convertase cleaves C5, after which C5b associates with C6 and C7 and inserts itself into the cell membrane. Upon binding of C8 and several C9 units, a lytic pore is formed: the MAC (5). Whilst the main cleavage products of complement ensure the downstream continuation of the pathway, the smaller cleavage products C3a and C5a are anaphylatoxins (3). Anaphylatoxins act as chemoattractant for immune cells and are important mediators in inflammation (11).

Next to its well-known role, it has become evident that intracellular complement plays an important role in homeostasis [reviewed in (12, 13)]. It has been shown that complement proteins are expressed by a large variety of cells that secrete these proteins into the local environment when needed. Recent studies also indicate that T cells not only express C3 but actually need intracellular C3 activation fragments for their survival (12, 13). Next, complement receptor CD46, described in more detail below, has also been identified as an important player in T cell homeostasis (14). Taken together, the complement system is well-orchestrated and highly effective in clearing invading pathogens. In addition, this system also plays an important role in activation of the adaptive immune system (11) and important for T cell homeostasis.

### Complement Regulators

The complement system is key in eradicating pathogens, but also plays an important role in clearance of for instance apoptotic or tumor cells. Due to the strength and omnipresence of the complement system regulation of this system is key to prevent damage to host cells (5, 15). However, complement dysregulation can be beneficial in certain circumstances. In retinitis pigmentosa complement overactivation on the levels of C3 results in enhanced phagocytic clearance of apoptotic photoreceptors by microglia resulting in limiting photoreceptor loss (16). In addition, the positive effect of complement activation on human cells was also shown for neuronal development (17). To ensure proper regulation of complement on host cells where needed, and to distinguish these from pathogens, complement regulators are in place. Most of these regulators of complement activity are encoded at the same locus on chromosome 1q32 and are formed by repeated complement control protein (CCP) domains (18). Up to date, a variety of soluble and membrane-bound complement regulators has been identified, which will be described here.

The soluble complement regulators can be found in plasma: Factor I (FI), Factor H (FH), C1-inhibitor (C1-INH), C4b binding protein (C4BP), clusterin (CLU) and vitronectin (Vn) (19, 20). FI is a protease specific for C3b and C4b, that can cleave these proteins in presence of a co-factor, forming their inactive counterparts iC3b and C4d. These co-factors are C4BP, FH and the membrane-bound complement receptor 1 (CR1) and CD46. By acting on C3b and C4b, FI can regulate all the complement pathways (21). Two soluble regulators act exclusively on the CP and LP: C4BP and C1-INH. C4BP prevents the formation of C3 convertase in the LP and CP, by binding to C4b. After C4BP binding of C4b, FI cleaves and inactivates C4b to C4d (19, 22). C1-INH has several roles in the suppression of inflammation and vascular permeability, but in the complement system it blocks the proteolytic activity of C1s, C1r and MASPs (23, 24). As a result, cleavage of C2 and C4 is inhibited resulting in less formation of the C3 convertase, C4bC2a.

FH is a regulator of the AP, next to its co-factor activity for FI. FH binds to sialic acid residues on host cells and to C3b, regulating the formation of C3bBb. FH can remove Bb from C3b, reversing convertase formation (25, 26). FH also has an alternative splice variant, Factor H-like 1 (FHL-1). FHL-1 consists of the first 7 CCP domains and is thereby able to fulfill the AP regulatory capacity similar to FH. However, as FHL-1 lacks the sialic acid residue binding domains of FH (CCP 19-20) its fails to distinguish effectively between host and foreign surfaces (27). FHL-1 CCP domain 7 is able to bind to sulphated glycosaminoglycans (GAGs) therefore, it is postulated that FHL-1 is mainly important in controlling the AP in local tissues such as the such as for example in the eye (28) Next to FH and FHL-1, humans also have five different Factor H-related proteins (FHR), which have arisen from duplication events of the *CFH* gene (29). These molecules share some domains with FH, but lack the complement inhibiting domains. Currently, there is no consensus on the precise function of these molecules *in vivo*, which is why they will not be discussed in detail here (30–32).

The last soluble complement regulators are CLU and Vn, both acting on the terminal complement pathway, inhibiting MAC formation. CLU binds to C7 to prevent membrane attachment, and can also bind C8 and C9 to prevent C9 assembly. Vn prevents the insertion of the MAC into the membrane by binding at the membrane-binding site of C5b-7 (19). Apart from their function as complement regulators, CLU and Vn have a range of cellular functions in lipid transport and cell adhesion respectively (22).

Next to the soluble complement regulators, five different membrane-bound regulators have been discovered that play an important role in regulation: CR1 (also known as CD35), membrane co-factor protein (CD46), decay accelerating factor (DAF, also known as CD55), CD59 and complement receptor immunoglobin (CRIg) (22). CR1 is a transmembrane protein that acts as a cofactor for FI and is a receptor for C3b, iC3b and C4b, inducing decay of C3 and C5 convertases (33). In this way, CR1 inhibits the CP, LP and AP. CR1 also plays a role in other immune functions, such as B and T cell regulation and phagocytosis and particle binding (34, 35). The other membrane-bound co-factor for FI is CD46, a transmembrane glycoprotein. Like CR1 it binds to the C3 and C4 fragments, but it does not have any decay activity, as opposed to CR1 (36, 37). CRIg is another transmembrane protein and an inhibitor of the AP. It is suggested to bind C3b, preventing C3bBb from cleaving C5. Unlike the other membrane-bound regulators that are widely expressed, CRIg is only present on a subset of cells: specific macrophages and dendritic cells (38, 39). Lastly, two other membrane-bound complement regulators are glycosylphosphatidylinositol- (GPI) anchored proteins CD55 and CD59 (3). CD55 accelerates the decay of the C3 convertases (22, 40). CD59, on the other hand, prevents the formation of the MAC by binding to C9 and preventing MAC insertion into the membrane (22, 40).

As the complement system needs to be regulated so tightly by a large number of proteins, it is to be expected that abnormalities in the complement system or the complement regulators are associated with a range of diseases. For many of these diseases, no adequate therapeutics are currently available, which is unsurprising considering that only two complement therapeutics are currently approved: anti-C5 antibodies (eculizumab and ravulizumab) and C1-INH (41, 42). Here, we will first describe the pathologies that complement regulators are involved in. Then, we will explore how the complement regulators can be used as potential therapeutics for these and other pathologies, and discuss relevant therapeutics currently in development. Altogether, this will give an insight in the significance and broad therapeutic applications for complement regulatory proteins.

## The Role of Complement Regulators in Pathologies

**Figure 2:**
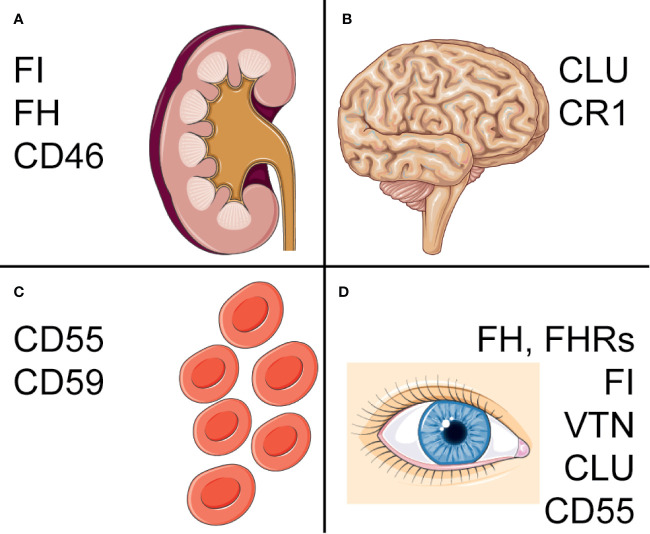
Complement regulators associated with pathologies. Overview of the major pathologies associated with complement regulators and polymorphisms or deficiencies associated with disease. **(A)** In the kidney, mutations in *CFH*, *CFI* and *CD46* are associated with aHUS, C3 glomerulopathy and IgA nephropathy. **(B)** Polymorphisms in *CLU* and *CR1* have been found to increase risk of AD. One of these *CLU* polymorphisms is also a risk factor for PD. **(C)** On erythrocytes, deficiencies of CD55 and CD59 on blood cells contribute to PNH, with isolated CD59-deficiency also associating with chronic hemolysis. **(D)** *CFH, CFI* and *VTN*, as well as a *CFHR1-3* deletion have been associated with AMD. A *CLU* polymorphism was found in pseudoexfoliation syndrome and Fuchs’ endothelial dystrophy. Lastly, a *CD55* polymorphism is a risk factor for pathological myopia. Figure designed using Servier Medical Art.

As described above, different cell types and tissues rely on different combinations of complement regulators and abnormalities in complement regulators are associated with a variety of pathologies (22). Amongst these are for example several red blood cell (RBC) mediated diseases, but also different cancerous malignancies and neurological diseases. The pathologies have been linked to both genetic and acquired abnormalities in complement regulators. Here, we will describe the different ways in which complement regulators have been linked to pathologies and focus on the diseases that are most well-studied. The most important polymorphisms and deficiencies are described in **Figure 2**. An overview of other diseases that have been linked to complement regulators, but are not discussed in detail here, are presented in **Table 1**.

**Table 1:** Additional pathologies where complement regulators were identified to play a role, that were not described in the text.

Pathology	Regulator	Association	Observations	Ref.
Allergic respiratory disease	CD55	Polymorphism	A CD55 SNP linked to decreased transcriptional activity, was associated with susceptibility to pollen and mite-induced respiratory allergies and enhanced specific IgE responses.	(43)
Geographic atrophy	CD46	Protein levels	Retinal pigment epithelial CD46 expression is altered early on in geographic atrophy development.	(44)
Traumatic brain injury	CR1 and CD59	Protein levels	CR1 and CD59 were significantly reduced in the first week after acute brain injury.	(45)
Recurrent miscarriages	C4BP	Polymorphism	C4BP mutations, of which some affecting expression level and FI co-factor activity, were found in women with recurrent miscarriages.	(46)
Cardiovascular disease	CR1	Polymorphism	CR1 polymorphisms are associated with increased risk of coronary artery disease.	(47)
	FH	Polymorphism	The FH Y402H polymorphism in FH was suggested to determine myocardial infarction susceptibility.	(48)
Sudden sensorineural hearing loss	FH	Polymorphism	The FH Y402H polymorphism has been associated with risk of sudden sensorineural hearing loss.	(49)
Idiopathic pulmonary fibrosis	CR1	Polymorphism	A CR1 polymorphism was associated with development of idiopathic pulmonary fibrosis.	(50)
Preeclampsia	CD46 and FH	Polymorphism	Two fetal CD46 SNPs were associated with preeclampsia, where placentas have increased complement deposition. An interaction with maternal FH and a C3 SNP might play a role as well.	(51)
Rheumatic disease	CD55 and CD59	Deficiency	CD55 and/or CD59 erythrocytes are present in a majority of patients with rheumatic disease.	(52)
Schizofrenia	Clusterin	Polymorphism	A clusterin polymorphism was associated with schizophrenia in Chinese patients with family history of disease.	(53)

### The Role of Complement Regulators in Anemic Pathologies

Many of the diseases in which aberrant complement regulation plays a role affect RBCs, which leads to hemolysis and anemia. As RBCs are continuously exposed to the complement system in the blood it is to be expected that they are highly vulnerable to complement-mediated lysis when their complement regulatory proteins are affected (54). The most well-described example of this is paroxysmal nocturnal hemoglobinuria (PNH). PNH is characterized by intra- and extravascular hemolysis and venous thrombosis, caused by the complement system. The disease arises from acquired somatic mutations in *PIGA* in hematopoietic stem cells, which encodes for phosphatidylinositol N-acetylglucosaminyltransferase subunit A, an essential enzyme in the synthesis of GPI-anchored proteins. As a consequence, GPI-anchored proteins are not present on cells arising from these stem cells, such as RBCs, platelets and neutrophils. Amongst these proteins are complement regulators CD55 and CD59, which cause the increased sensitivity to complement-mediated hemolysis (55–57).

Currently, the only treatments approved for PNH are eculizumab and ravulizumab, both C5 monoclonal antibodies, inhibiting C5a generation and formation of the MAC. Eculizumab is able to reduce intravascular hemolysis, fatigue and transfusion requirements in PNH patients, although responses are variable between patients (58–60). The variability of responses can in some cases be explained by a C5 mutation that prevents eculizumab binding (61). However, the main variation arises from the fact that eculizumab does not prevent C3b deposition on RBCs. Opsonization by C3b induces removal of the RBCs by phagocytosis, which leads to an increase in extravascular hemolysis as shown in PNH patients receiving eculizumab treatment (62). Susceptibility to extravascular hemolysis upon eculizumab treatment has been linked to a CR1 polymorphism. Decreased CR1 on the RBC surface would lead to a decrease in C3b decay and thus for increased opsonization that can induce extravascular hemolysis. *CR1* has two co-dominant alleles that lead to either high (H) or low (L) expression of the regulator respectively. It has been shown that PNH patients with a H/L or L/L genotype have a lower expression of CR1 and require more transfusions (63). Although it has been shown that compstatin is a highly effective inhibitor of C3 that can prevent extravascular hemolysis (64, 65), it is not FDA approved yet (66).

Next to the combined CD55- and CD59 deficiency in PNH, a few isolated CD55- and CD59 deficiencies have been described. Isolated genetic CD59-deficiencies have been associated with chronic hemolysis like PNH, as well as with other symptoms such as neuronal damage and recurrent strokes (67, 68). Interestingly, RBCs specific isolated deficiencies of CD55 do not seem to induce hemolysis. A small group of patients has been found to have CD55-deficient RBCs, named the Inab phenotype. These individuals do not have any specific complaints and have a variety of CD55 silencing mutations (69, 70). Although *in vitro* research showed an increase in C3b deposition on the RBCs of this phenotype compared to healthy cells, an increase in hemolysis was not found (71). Not all of these patients are however fully deficient in CD55, which *in vivo* might prevent some complement-mediated activity on RBCs. Furthermore, a CD55 loss-of-function mutation causes an autosomal recessive disorder characterized by hyperactivation of complement, angiopathic thrombosis, and protein-losing enteropathy (CHAPLE) syndrome. Here, the lack of CD55 leads to overactivation of the complement system, which leads to gut inflammation induced by anaphylatoxins in these patients (72–74). Interestingly, again increased hemolysis was not reported in any of the CD55-deficient patients, which could be an indication that CD59 is more important in complement regulation on RBCs. The important role of CD59 is further illustrated by the fact that CD59-deficient RBCs are highly sensitive to complement-mediated lysis. However, RBCs of a PNH patient showed the highest sensitivity for complement mediated lysis compared to the RBCs with a single complement regulator deficiency (75).

Hereditary hemolytic anemias are a group of anemias that share the common feature of decreased RBC survival leading to anemia, which is caused by inherited disorders in certain membranes, enzymes or hemoglobin. Examples are hereditary spherocytis, caused by membrane protein deficiencies, congenital microcytosis, caused by defects in hemoglobin (76) and sickle cells disease, caused by a hemoglobin mutation inducing deformation of affected RBCs under stress conditions, that leads to anemia in homozygous patients (77). The expression of membrane-bound complement regulators CD55 and CD59 in patients with hereditary spherocytis and congenital microcytosis has been investigated. FACS analysis showed a decrease in CD55 expression on erythrocytes of both patient groups compared to controls. However, immunoblot showed a less clear difference. CD59 was unaffected in both patient groups indicating that the decrease in CD55 expression is not caused by a *PIGA* mutation, similar to PNH patients, as a GPI anchor defect would affect both CD55 and CD59. It remains debatable whether low CD55 expression contributes to the anemia in these patients as a correlation between the expression of CD55 and severity of the anemia was not found and the effect on complement-mediated RBCs destruction was not studied (78). Like in the before mentioned anemias, differential expression of CD55 and CD59 on erythrocytes was also found in sickle cell disease patients. Although these patients expressed decreased CD55 and CD59 expression on RBCs, this could not be linked to disease severity, suggesting that these regulators do not play a significant role in the pathophysiology of hemolysis in sickle cell disease (79). Recent research does however suggest a role for complement activation in sickle cell disease pathophysiology, where overactivation of complement has been found on sickle cell erythrocytes compared to healthy erythrocytes (80). Altogether, these studies indicate directions for future research into the mechanistic role of complement regulators and complement activity in hereditary hemolytic anemias to determine the exact role of complement regulators in these pathologies.

### The Involvement of Complement Regulators in Renal Disease

Complement is differentially regulated in the kidney compared to other tissues. The glomerular basement membrane is very sensitive to complement damage and relies completely on soluble complement regulators, as it is directly exposed to blood and the complement system, unlike most other basement membranes (81, 82). It is therefore understandable that different kidney pathologies have been associated with anomalies in soluble complement regulators, which results in overactivation of the complement system (83).

Atypical hemolytic uremic syndrome (aHUS) is a kidney disease that can have serious complications, with a mortality rate of 25% without eculizumab treatment (84). Characterized by microangiopathic hemolysis, thrombocytopenia and renal failure, it is caused by disbalance between regulation and activation of the AP. This disbalance is caused by decreased activity of complement regulators, or increased activity of complement proteins. Many aHUS cases have an identified genetic basis. Mutations linked to aHUS have been identified in several complement-related proteins, of which mutations in FH, FI, CD46 and C3 are found in approximately half of the patients. These mutations often lead to altered function or expression (85–87). More rarely, mutations in C2 and FB are found in aHUS patients (83, 88). In around 10% of the patients, autoantibodies against FH have been found. Most of the FH mutations as well as the FH autoantibodies target FH CCP 20. As FH CCP 19-20 is most important for FH binding to GAGs predominantly expressed in the glomeruli basement membrane, it might not come as a surprise that exactly these mutations and antibodies are linked to aHUS (89–91). Current treatment of aHUS consists of either plasma infusion or exchange, or eculizumab. Plasma infusion and exchange can replenish complement regulators in the blood of the patient and is especially beneficial in patients with FH and FI mutations (92). Eculizumab is now the preferred treatment, as it prevents complement-mediated damage as described above for PNH and is more safe than plasma transfusions (84).

Next to aHUS patients, C3 glomerulopathy patients suffer from dysregulation of the AP as well. In contrast to aHUS patients, these patients are characterized by C3 deposits in glomeruli, which leads to disruption of kidney function, but the disease is not associated with thrombocytopenia, anemia, or other systemic involvement (93). The pathophysiology of disease is not always clear, but in some cases it is known to be caused by autoantibody formation against C3bBb, which stabilizes the convertase and thus increases its activity, or by mutations in C3 or the complement regulatory proteins FH or FI (94–96). Another kidney disease where an FH polymorphism increases susceptibility is IgA nephropathy, a form of glomerulonephritis where C3 depositions are found on affected kidney tissue, together with IgA. The FH polymorphism identified for IgA nephropathy has not been linked to aHUS. A meta-analysis showed significant association between this minor allele and IgA nephropathy risk (97). This strengthens the hypothesis that IgA nephropathy is caused by overactivation of complement, although no functional implications of this specific polymorphism have been described.

Lastly, chronic kidney disease (CKD) is a condition where decreased kidney condition takes place over a longer period of time, regardless of the underlying cause. Although the pathologies mentioned above can cause CKD, the majority of cases is caused by diabetes and hypertension (98). Anemia is found in almost half of the CKD patients, being more prevalent in end-stage disease (99). A study of anemic CKD patients investigated CD55 and CD59 expression and showed that patients had an altered expression of CD55 and CD59 on RBCs, with more CD55- and CD59-deficient RBCs in patients than in healthy controls. CD55- and CD59-deficiency would make RBCs more susceptible to complement-mediated hemolysis. Increased hemolysis was confirmed in patients with high levels of CD55- and CD59 deficient RBCs. The underlying cause of CKD in these patients was not reported, making it difficult to speculate on the mechanisms involved (100). Furthermore, *VTN* has been shown to be upregulated in chronic kidney disease. This is possibly related to the fact that it functions as a ligand for the urokinase-type plasminogen activator receptor. This receptor is not found on healthy kidney cells, only getting expressed in diseased kidney cells, where it is involved in cell recruitment and migration (101). Up to date, no studies have been conducted that assess the role of Vn on complement regulation in CKD.

### Complement Regulators in Diseases Affecting the Eye

Complement activation in the eye is highly reliant on local production of complement proteins, due to its anatomical characteristics (102). The inner part of the eye is shielded off by a sheet of extracellular matrix called the Bruch’s membrane. Like the glomerular basement membrane in the kidney, it is directly exposed to circulation and thus to complement activity, making it vulnerable to dysfunctional complement regulators (103). Bruch’s membrane is impermeable to larger proteins, these thus need to be locally produced. This also holds for the larger proteins of the complement cascade, such as C3b, FH, FB, and FI. It has been shown that FHL-1, a truncated splice variant of FH, is small enough to diffuse over Bruch’s membrane and could thus play an important role in the eye (28). The importance of complement in the eye has been illustrated in age-related macular degeneration (AMD). As one of the leading causes of blindness AMD is affecting more than 5% of the elderly above 75 years old, for which the role of complement in disease has been widely studied.

AMD is marked by progressive destruction of the macula, as the retinal pigment epithelial cells that normally maintain retinal homeostasis are unable to do so. Currently, AMD is considered to be caused by a combination of genetic and environmental risk factors. Different complement genes have been associated with AMD, which can altogether account for 40–60% of the heritability of the disease. Amongst these genes are *CFH*, *CFI* and *VTN* which indicates that a dysregulation of complement activation is at play here.

Several mutations were found for *CFH*, amongst which the common Y402H variant, and were all associated with decreased protein expression or functionality of FH and FHL-1 (104, 105). Similarly, the *CFI* mutations found were associated with decreased serum levels of FI, although the effect of these polymorphisms on FI production within the eye is not known (106). For the *VTN* polymorphism identified no functional consequences have been described yet (107). Lastly, serum levels of FHR-4 were increased in AMD patients compared to healthy controls, while complete *CFHR1* and *CFHR3* deletion has been described to be protective against AMD (32, 108). While FHR-3 is locally produced in the retina, FHR-4 expression has not been demonstrated here yet (109, 110). It has been suggested that FHR-4 might inhibit FHL-1 functioning in AMD, as competition assays showed that FHR-4 outcompetes FHL-1 in C3b binding (32).

Pseudoexfoliation syndrome is a pathology characterized by the deposition of abnormal fibrillar extracellular material in both non-ocular and ocular tissue, which can lead to glaucoma (111, 112). CLU is reduced at both mRNA and protein levels in several parts of the eyes of pseudoexfoliation syndrome patients compared to healthy controls (113). Under stress conditions CLU would be conventionally expected to be increased: amongst its biological functions are its role as an extracellular chaperone, which inhibits stress-induced precipitation and the aggregation of misfolded proteins (114). Thus, its downregulation here could be a contributing factor to the accumulation of fibrillar extracellular material (113). A polymorphism in *CLU* might contribute to genetic risk of pseudoexfoliation syndrome, but was not relevant when the age of controls was corrected for, and was not significant in logistic regression. Thus, common genetic variation in the CLU gene does not seem to play a major role (115). Next to the involvement of CLU in pseudoexfoliation syndrome, C3 was found to be significantly upregulated in aqueous humor in eyes of patients with pseudoexfoliation syndrome indicating an important role for complement activation during this disease (112). The mechanisms behind the role of complement in pseudoexfolation syndrome have not been elucidated yet, calling for more research to indicate whether other complement regulators might be implicated in its development.

Furthermore, a *CLU* polymorphism was associated with Fuchs’ endothelial dystrophy, which is a degenerative disease of the corneal endothelium. This polymorphism results in overexpression of CLU in the corneal endothelial cells of these patients. It was hypothesized by the authors that this specific polymorphism in the *CLU* gene might affect its secretion by corneal endothelial cells, which would expose these cells to physiological stress (116). Lastly, a polymorphism in CD55 was repeatedly associated with the risk of development of myopia. Myopia, more commonly known as nearsightedness, is the leading cause of vision loss in young people, stemming from excessive axial elongation of the eye (117, 118). The effect of this polymorphism on CD55 function or expression has not been described yet. Complement activity as measured by C3, C4 and CH50 activity in serum is increased in pathological myopia patients, which could be an indication that dysregulation of complement is at play here (119). However, as measurements of complement activity in serum are not proper indications of complement activity in the eye, care should be taken in concluding complement plays a role in myopia patients, for which further research is needed.

Taken together, there are multiple strong links between mutations in complement regulatory proteins and pathology in eye diseases. Studying complement activation and regulation within the eye is difficult, limiting our current knowledge. Although some *in vitro* models have been developed, the current *in vivo* models are still insufficient to mimic complement activity in the human eye (102, 105, 120, 121). Knowledge considering the effect of the currently identified polymorphisms in the eye, could improve our understanding of the pathology and aid in the development of therapeutics.

### Complement Regulators in Neurological Disease

The blood–brain barrier is largely impermeable for plasma proteins, like the complement proteins. As only a fraction of complement proteins passes the blood–brain barrier and there is minimal local production, the healthy brain expresses low levels of complement proteins. In diseased brain however, the blood–brain barrier can get disrupted and local production can be increased, contributing to a rise in complement activity. If complement is activated in the brain, the brain’s glia cells and neurons have a low tolerance for it, due to low levels of complement regulators (122). The role of complement in diseases of the central nervous system has become more apparent in recent years. It has been shown to play a role in brain development, acute brain trauma and neurogenerative disease (123). Here, we discuss the links between complement regulators and Alzheimer’s disease (AD) or Parkinson’s disease (PD) as the role of regulators here has been most well-studied and the diseases largely impact global health.

AD is a common neurodegenerative disease, with almost 30 million cases worldwide in 2019, that leads to loss of cognitive function, caused by neuropathologic lesions in the brain (124). These lesions can be caused by amyloid plaques or neurofibrillary degeneration. Complement has been shown to contribute to the inflammation of the brain that occurs in AD, and to be activated in and around the amyloid plaques (122). The overactivation of complement contributes to disease progression, resulting in synapse loss and neurodegeneration (125). The evidence for the association of complement regulatory genes with AD has been given multiple times. Polymorphisms in both *CR1* and *CLU* have been repeatedly demonstrated to increase risk of AD (126–129). *CLU* has been shown to be upregulated in the AD brain, as well as to play a role in the clearance of β amyloid, which is the major component of the amyloid plaques (124). How this upregulation of *CLU* in the AD brain affects local complement activation has not been investigated. The functionality behind *CR1* polymorphisms in AD have not been fully elucidated either. One of the risk-increasing *CR1* polymorphisms results in a single amino acid change that has been associated with mildly increased sCR1 levels and binding affinity for C1q (130). The other is an intronic mutation associated with increased length of the final protein by duplication, resulting in more C3b binding sites which could increase CR1 effectivity (122, 131). However, this variant is also associated with decreased expression levels. Overall, it seems that AD patients with the long CR1 isoform show an progressively decreased expression of CR1 on cells, thus reducing the total effectiveness of CR1 (132). Future research should indicate what the effects of the found *CR1* and *CLU* polymorphisms are on protein levels and functionality within the brain.

PD is another common neurodegenerative disease, which is also characterized by protein depositions in the brain. Research on the brains of PD patients has repeatedly shown complement activation in affected tissues (133, 134), and the dysregulated complement system has been suggested to play a role in pathology of PD (123). This was reason to investigate the abovementioned found polymorphisms for AD in PD. Only one of the *CLU* polymorphisms found for AD also showed to be a risk factor for the development of PD (135). However, levels of CLU in cerebrospinal fluid and plasma were not found to differ between patients and healthy controls. A correlation between CLU levels and amyloid- ß42, T-tau and P-tau levels was found, which are proteins that play a role in both AD and PD. Altogether these results suggest a direct interaction between amyloid- ß42, tau and CLU (136). It might not be surprising to see that CLU is associated with several neurological diseases, as it has been implicated in neuronal synapse function (53). Further research should indicate whether CLU also contributes *via* its role as complement regulatory protein in this system.

### Complement Regulators in Oncology

The complement system has become strongly implicated in different types of cancer in recent years, but its role in oncology has not been well defined (137). In fact, the role of complement in carcinogenesis, has been described as a dual-edged sword (138). The dual mechanism of complement in carcinogenesis could be similar to that of inflammation, in which complement is a large contributing factor. Initially, acute inflammation can cause early detection and clearance of malignant cells, but chronic inflammation might also contribute to the spread of malignant cells (139). Specifically, the anaphylatoxins C3a and C5a have been described to contribute to an inflammatory tumor microenvironment. Their production upon local activity of the CP and AP on the tumor creates an environment that favors tumor growth and metastatic spread, contributing to tumor progression (137, 139–141). This role for C3a and C5a has been shown in different cancer types, in the form of promoting angiogenesis, inhibiting anti-tumor immune responses and by triggering tumorigenic, survival and anti-apoptotic pathways in tumor cells (141).

Although the importance of complement in cancer has been demonstrated, its function and overall effect have not been fully elucidated. The currently available literature on complement regulators in cancer specifically is limited and sometimes contradictory. This could in part be explained by the fact that only few human studies are available, with most of the data stemming from animal models or *in vitro* studies (137). In addition, complement regulators do not only have impact on the complement system, but also play an important role in the adaptive immune system by regulating and activating T-cells (142), making studies regarding their role extremely difficult. In general, it is believed that high expression of complement regulatory proteins is linked to worse clinical outcome (142). Here, we will give a brief overview of the currently available human studies of complement regulators in cancer.

Colon cancer is the second-most lethal cancer in the world (143). Although no complement regulator polymorphisms for risk of colon cancer have been identified, CD46, CD55, and CD59 were all upregulated in colon cancer compared to adjacent healthy tissue (144, 145), with CD55 and CD59 expression correlating with tumor stage and differentiation level (146). In addition, patients with an increased CD55 expression in their tumor tissue had a worse 7-year survival rate, with tumors with high levels of CD55 more aggressive than tumors with low CD55-levels (147). Patients with colorectal cancer also showed increased soluble CD55 in stool specimens compared to healthy controls and to patients with other gastrointestinal disease. Whether the soluble CD55 was secreted as such, or whether it arose from cleaved membrane-bound CD55, was unclear (148). These studies combined suggest that the increased expression of complement regulators protects malignant colorectal cells from being attacked by the human immune system, especially for CD55. A mouse xenograft study has shown that a blocking CD55-antibody inhibits tumor growth and increases survival of mice. *In vitro*, the combination of a blocking CD55-antibody with regular chemotherapy has a synergistic effect (149). This therapeutic effect suggests a clinical relevance for CD55 in colon cancer.

Another common and deadly cancer type is non-small cell lung cancer (NSCLC). NSCLC has been associated with the complement system in multiple ways, but the precise mechanisms in place have not been elucidated yet (150). On the genetic level, both a CD55 and a CR1 polymorphism have been associated with risk of NSCLC development (138, 151). The CD55 polymorphism found in NSCLC is associated with decreased transcriptional activity of CD55. This was a surprising finding, as complement regulation was initially expected to be increased on malignant cells, protecting them from detection by the complement system (151, 152). There might be a differential effect of CD55 between healthy and cancer cells at play here, as CD55 in NSCLC is not only down-regulated, but also sialylated. Possibly, sialylated CD55 can execute its function longer than regular CD55, as it has a high resistance against proteolysis and can thus be retained on the cell surface for a longer time (153). Another explanation could be that NSCLC tissue does not require complement regulation as much, as the bulky tissue formation ensures that not all cells are exposed to the alveolar cavity, and thus to complement activity (153). The CR1 polymorphism found in NSCLC was not further functionally described. Furthermore, at the protein level *in vivo* xenograft study in mice showed that FH expression is critical for NSCLC tumor growth. Whilst NSCLC cells *in vitro* were shown to be insensitive to complement-mediated lysis, even largely when FH was blocked, blocking FH *in vivo* significantly decreased xenograft size (154).

Apart from the solid tumors described above, some research is available that links different types of leukemia and lymphoma to complement regulators. In a group of leukemia patients, comprised of patients with acute myeloid leukemia, acute lymphoblastic leukemia and chronic lymphocytic leukemia, soluble CR1 was found in high concentrations, regardless of specific leukemia type (155). The soluble CR1 originates from cleaved and shed membrane-bound CR1. This soluble CR1 maintains its complement regulatory activity, allowing for complement regulation in serum. Although it seems unlikely that this would lead to reduced overall complement activity in plasma *in vivo*, it could be that soluble CR1 shedding leads to local down-regulation of complement activity at sites of tumor infiltration, which could aid the malignant cells locally (155). This regulating characteristic of soluble CR1 has also been demonstrated in other studies, and soluble CR1 is now used to therapeutic advantage in complement-mediated diseases (156–158) as discussed below. The effects found for soluble complement regulators could be caused by their effects on the tumor microenvironment. The tumor microenvironment is the total of cancer cells, stromal cells and extracellular components around the tumor, where complement has been shown to play a regulatory role to which soluble regulators might contribute (159, 160).

The last type of tumor to be described here is non-Hodgkin’s lymphoma. Non-Hodgkin’s lymphoma is the most common type of lymphoma, with around half a million new cases a year and diffuse large B cell lymphoma and follicular lymphoma as its most common subtypes (161). In follicular lymphoma, a CD46 polymorphism is associated with decreased event-free survival, while the CD55 and FH polymorphisms are associated with increased event free survival. In diffuse large B cell lymphoma, a possible association with increased event-free survival was found for a *CLU* polymorphism (162). Unfortunately, there are no studies available linking any of these polymorphisms to expression levels or functionality of the protein, and there is no consensus on the role of complement in these diseases. A more recent study acknowledged that the role of complement in lymphoma is complex, but could indicate FHR-3 as a biomarker for the response to specific immunotherapies (163).

In conclusion, both solid malignancies and blood cancers have been associated with complement regulators. Here, we have described a selection of cancer types related to complement regulators, based on the extent to which they have been studied in humans. Based on this research, it is not possible to be conclusive on the role of complement regulators in cancer. The current indications of the many polymorphisms in complement regulators involved in cancer, can be used as directions for future research into the mechanisms and purposes of complement regulation in cancer.

Overall, we have demonstrated the broad range of diseases in which complement plays a role, giving examples from RBCs, the eyes, kidney, brain and oncology. In addition, **Table 1** shows other pathologies found linked to complement regulators in the current research. Altogether, the great amount of pathologies linking to dysfunction of complement regulators implies that future therapeutics based on complement regulators could potentially be applied widely. Next, we will explore the potential of such therapeutics currently available and under development.

## Future Potential of Utilizing Complement Regulators in Therapeutics

As discussed above, a broad range of pathologies arises from dysfunction of complement regulation, leading to overactivation of the complement system on healthy cells, or insufficient complement activation on malignant cells (22, 137). Currently, only two therapeutics are approved to target the complement system, eculizumab/ravulizumab and C1-INH, which is insufficient to treat the wide range of complement-related pathologies. Other complement-targeting therapeutics are currently in development, such as compstatin, and FB and FD inhibitors (42, 164). The clinical success of these drugs has been limited to this date. The anti-C5 antibody eculizumab, as mentioned previously, is currently approved for diseases such as PNH and aHUS. When effective, eculizumab treatment increases patients’ life expectancy to that of healthy person (165, 166). However, the drug is not effective in all patients (165, 166). Around 15% of aHUS patients do not respond to treatment, as well as 3% of PNH patients, with 30% of treated patients still requiring regular blood transfusions (61, 167, 168). In AMD, a phase III trial with eculizumab and a similar trial with an anti-FD antibody did not show a slowing effect on geographic atrophy development (169, 170). Together, this data indicates that although the fact that complement plays an important role in AMD, complement modulation seems a challenging and uncertain therapeutic option. Lastly, compstatin is a small cyclic peptide that binds C3 and inhibits convertase formation and C3 cleavage, of which two analogs have been clinically evaluated: AMY-101 and APL-2. To this date, AMY-101 has only been tested in a phase I trial, with phase II trials still expected to start (66). APL-2, a compstatin variant with an increased half-life due to adapted pegylation, has been successfully tested in PNH patients in phase I studies, with a phase III study on its way (66, 83).

**Figure 3:**
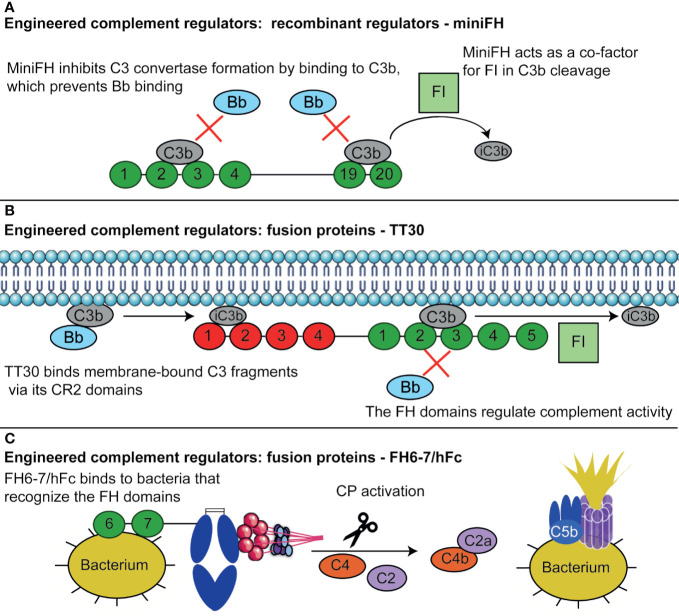
Therapeutics based of complement regulators. **(A)** MiniFH has been designed from FH CCPs 1-4 and 19-20 to open up an extra C3b binding domain. This means it can bind two C3b molecules simultaneously, where regular FH can only bind one. MiniFH inhibits C3bBb formation and exert FI co-factor activity. **(B)** TT30 is a fusion protein of the first 4 domains of CR2 and the first 5 domains of FH. The CR2 domains recognize tissue-bound C3 fragments, while the FH domains regulate complement activity. Thus, the protein is targeted towards tissues where complement is already activated, where the FH domains can then exert complement regulatory activity. **(C)** FH6-7/hFc contains FH CCP 6 and 7, and the Fc part of an antibody. Certain bacteria bind FH *via* CCP FH6-7, normally to evade the complement system. These CCPs are utilized here to target these bacteria, upon which the hFc induces CP activation by C1q binding resulting in complement activation *via* the CP finally resulting in opsonization and lysis of bacteria. Figure designed using Servier Medical Art.

Finding targets for complement therapeutics is not easy, as it is often unclear which of the many complement components is involved in the pathology of disease and the functions of the system are broad, meaning that side-effects of drugs interfering with the complement system are likely to occur. Furthermore, the plasma concentrations and turnover rate of the complement proteins are high and their production occurs at different sites in the body which can complicate drug delivery (4). The field of complement therapeutics has been evolving to solve these challenges, looking at complement regulators to prevent side-effects of targeting complement systemically. Here, we discuss therapeutics based on complement regulators currently in the pipeline, and future possibilities to use the mechanisms of complement regulators in therapeutics (**Figure 3**).

### Plasma-Derived and Recombinant Full-Length Complement Regulators

Full-length complement regulators can be therapeutically used to aid patients with deficiencies functional defects in their regulators. The best example of this approach is the use of C1-INH in hereditary angioedema. Hereditary angioedema is caused by a genetic C1-INH deficiency which can result in life-threatening edema from excessive bradykinin release (171, 172). The plasma-derived C1-INH can prevent angioedema attacks completely, providing a safe treatment option with no significant side effects (171, 173). Additionally, C1-INH has been recombinantly produced from the milk of transgenic New Zealand white rabbits (174). The recombinant and plasma-derived C1-INH did show comparable activity in hereditary angioedema, but the half-life of recombinant C1-INH was much shorter (171). This might pose a problem when using recombinant C1-INH for complement mediated diseases such as ischemia–reperfusion injury, acute myocardial infarction, autoimmune hemolytic anemia and renal transplantation (175–178).

Another complement regulator for which both a plasma-derived and a recombinant variant is in development is FH. As described, mutations of FH are clearly linked to development of complement-mediated renal diseases. As described above for aHUS, patients with functional FH mutations can be treated with plasma infusions. However, plasma infusions are not without risks, as they can lead to allergic reactions or fluid overload (179). Plasma-derived or recombinant FH could potentially be more purposeful in supplementing mutated non-functional FH. FH-deficient mice, mimicking C3 glomerulopathy, have been treated with human plasma-derived FH, resulting in a reduction in complement activity and C3 deposition on the glomerular basement membrane (180–182). Recombinant FH has been produced in insect cells, the yeast *Pichia pastoris*, mammalian HEK and COS7 cell lines (91, 183, 184). Although the produced proteins were biologically active, success was limited by low yield and aberrant glycosylation patterns. Currently, the most successful production of recombinant FH with full human glycosylation pattern has been achieved in the moss *Physcomitrella patens* (FHmoss) (185, 186). FHmoss has full biological activity and was shown to reduce complement activation and hemolysis in aHUS patient sera. Experiments in FH-deficient mice treated with FHmoss showed a reduction of glomerular C3 accumulation and AP activity. These experiments also showed that FHmoss is cleared from the circulation faster than plasma-derived FH and could not be detected anymore in plasma after 6 h, which is a potential obstacle for the clinical use of FHmoss (185). The success of the *P. patens* system has been further illustrated by the production of MFHR1, which is a recombinant regulator with domains from FH and FHR-1. This is a biologically stable protein that has an even stronger *in vitro* regulatory activity on the AP than FH (187).

Next to the fluid-phase regulators C1-INH and FH, recombinant soluble variants of membrane-bound regulators have been developed. While soluble CR1 (sCR1), CD46, CD55 and CD59 were all produced and tested successfully *in vitro* (188–190), only sCR1 has progressed to clinical trials (158). With regards to sCR1, two highly similar recombinant molecules have been designed (TP10 and TP20). Both are effective inhibitors of C3 and C5 convertases and were shown to prevent complement-mediated tissue damage in different animal models of vascular injury (158, 191). TP20 is a derivative of TP10 that contains Sialyl-Lewis X tetrasaccharide (sLe^x^) groups to target it towards the endothelium *via* binding of E- and P-selectin receptors. This modification has made it more effective in *in vivo* models of cardiovascular and lung disease than TP10 (191, 192). Recently, TP10 has been clinically evaluated in several indications requiring cardiopulmonary bypass, as complement activity has been shown to play a role in the inflammatory response after acute myocardial infarction. Intravenous administration of TP10 reduced complications and mortality in male patients undergoing cardiopulmonary bypass, but not in female patients, although this gender difference was not caused by the complement suppressing effects of sCR1 (156). In lung transplantations, TP10 administration led to a shorter intubation time, although other clinical outcomes were not affected by TP10 (156). Taken together, use of full length complement regulators seems an efficient strategy. Use of plasma-derived proteins is limited due to the availability of plasma, while recombinant proteins struggle with a short half-life. In addition to the proteins described here, we could imagine the use of full length FI to be further explored, as it has been shown that plasma infusions are also successful in aHUS patients suffering from a mutation in FI (92, 193).

### Engineered Complement Regulators

Apart from the variety of full-length complement regulators researched for therapeutic usage, engineered complement regulators are also under development. These proteins are recombinant complement regulators, but rather than being designed to mimic the human regulator as closely as possible, they are adapted to enhance their therapeutic effects (194). For FH, two engineered variants have been designed: miniFH and midiFH. MiniFH contains CCP 1-4 and 19-20 of FH, whilst midiFH is its duplicated variant, coupling two miniFH molecules with a short linker (**Figure 3A**). MiniFH was designed specifically to enhance C3b binding activity, as the CCPs masking the C-terminal C3b binding domain in full-length FH are deleted. Both proteins are more effective in decay accelerating activity than FH and FHL-1, but less effective in FI co-factor activity (195, 196). MidiFH outcompetes miniFH in the inhibition of AP activation, which can probably be explained by its extra C3b binding sites (195). Engineered variants of FH could be relevant in the treatment of PNH as *in vitro* studies already showed that both miniFH and midiFH are effective in protecting PNH erythrocytes from complement-mediated lysis (195, 197). The efficacy of miniFH could even be improved by introducing the dimerization domain of FHRs in the protein, which lead to the formation of homodimers and prolonged its half-life. These homomeric miniFHs have been tested *in vivo* in FH-deficient mice, where they showed a reduction in glomerular accumulation of C3 (198). Taken together, engineered complement regulators such a miniFH show promising results both *in vitro* as well as *in vivo*. To date, Amyndas has engineered a mini FH for which phase-1 clinical studies for treatment of AMD are planned (42).

A different approach to engineered complement regulators is the design of fusion proteins to optimally utilize their regulatory activity. Currently, fusion proteins have been described containing (parts of) CR1, CD55, CD46, and FH to target the AP, CP and/or the LP. Mirococept is a fusion molecule containing the first three CR1 SCRs, containing the complement regulatory activity, followed by an amphiphilic peptide that increases its affinity for cell surfaces. *In vitro*, Mirococept can inhibit C3 and C5 convertase activity similar to CR1 (199). Mirococept is also beneficial *in vivo*, which was demonstrated using rat models for intestinal ischaemia and reperfusion injury and for kidney transplantation (199, 200). Mirococept was shown to be safe in a phase I clinical trial, with a phase II clinical trial in kidney transplantations currently underway (201). To the best of our knowledge, the results of this trial have not been published yet (202).

TT30 is an FH and CR2 fusion protein that has been designed to selectively inhibit the AP locally at activation sites in order to minimize the risk of infection or autoimmune disease which could increase upon systemic complement inhibition (**Figure 3B**) (203). To this end CR2 domains were used, as CR2 only binds to the tissue-bound C3b breakdown products iC3b, C3dg, and C3d, thereby specifically acting at sites of complement activation on tissue. TT30 contains the first four CCPs of CR2, which bind to the C3b breakdown products, and the first five domains of FH, which regulate AP activity. *In vitro*, TT30 is effective in both inhibiting the C3 and C5 convertase. Next, subcutaneous administration of TT30 in cynomolgus monkeys showed almost complete inhibition of the AP, lasting up to 24 h, and partial inhibition of the CP (203).

The above-mentioned fusion proteins utilize CR1 and CR2, which mainly target the AP. Although the role of the AP has been well described in diseases as PNH and aHUS, it is not the only complement pathway to think of when aiming to target the complement system with therapeutics. Recently, fusion proteins to target the LP were developed and suggested to have potential in the treatment of ischemia–reperfusion injury and delayed kidney graft function (204). These proteins consist of the first domains of CR1, CD55 or FH with MAP-1 or sMAP in order to inhibit LP activation at the C3 level and prevent MAC formation. MAP-1 and sMAP are alternative splice variants of MASP-1 and MASP-3 respectively, lacking the proteolytic site, thus binding to the pattern recognition molecules of the LP without inducing subsequent C2 and C4 cleavage (7, 205). MAP-1:CD55^1-4^ and MAP-1:CD35^1-3^ effectively inhibit the LP in *in vitro* assays that measured deposition of C3, C4, and MAC on mannan. The effects of MAP-1:CD55^1-4^ were stronger, especially at the terminal pathway level. The root of this difference could not be pinpointed, although it was suggested to be caused by the absence of certain CD35 domains in MAP-1:CD35^1-3^ that are normally involved in its co-factor activity (205). A different study fused full-length murine MAP-1, murine sMAP or human sMAP to the same FH domains. The murine MAP-1 FH fusion protein, MAp44-FH, showed only limited activity both *in vitro* and *in vivo*, while the murine sMAP-FH inhibited the activity of both the AP and LP. Human sMAP-FH showed an inhibitory effect on the AP and LP in human serum as well, which was stronger than the effect of the sMAP or FH domains separately (206). The stronger effect of sMAP-FH than of MAp44-FH could possibly be explained by their competition with MASPs to form complexes with the pattern recognition molecules. sMAP competes with MASP-2 for binding, while MAp44 competes with MASP-1 and -3. MASP-1 is 13.6 times more abundant in serum than MASP-2, which results in MASP-1 outcompeting MAp44-FH in serum at the studied concentrations, which were constant between MAp44-FH and sMAP-FH (206). To conclude, several LP inhibiting complement regulators have been engineered, of which the FH domain also targets the AP. The AP and LP both contribute to diseases as AMD and ischemia–reperfusion injuries, were MAP-1:CD55^1-4^, MAP-1:CD35^1-3^, and sMAP-FH could potentially aid in the treatment of such pathologies.

The last fusion protein composed of two different complement regulators to be described here is decay-cofactor protein (DCP), a fusion product of CD55 and CD46 (207) that was designed to target both the AP and CP. Different CCPs of CD55 and CD46 were combined into fusion proteins that would retain their inhibitory effect on CP and AP activation. A fusion protein of CD55 CCP2-3 and CD46 CCP3-4 showed the largest inhibitory activity *in vitro*, upon which DCP with maximal inhibitory effect was achieved by selecting the most effective mutant (207). Although successful inhibition of the AP and CP could be achieved *in vitro*, this protein has to this date not been tested *in vivo*.

Next to the fusions of complement proteins with the regulators, other fusion proteins contain (parts of) antibodies. Three different types of regulators fuse parts of antibodies with a complement regulator, which have been developed to target myasthenia gravis, the AP in several pathologies or bacterial infections respectively. Firstly, the fusion protein designed to treat myasthenia gravis contains CD55 and an antibody fragment. Myasthenia gravis is an autoantibody-mediated disease, where damage to neurons at the neuromuscular junction is enhanced by complement activity (208). The fusion protein to treat this disease consists of an scFv antibody fragment targeting the nicotine acetylcholine receptor, that did not affect ion channel function, and CD55, which would regulate complement activation on the neuron surface. This fusion protein induced a reduction in complement activation *in vitro* and reduced clinical severity *in vivo* (208, 209). Secondly, a fusion protein of CRIg and Fc of a murine IgG1 (CRIg-Fc) is a soluble version of CRIg and was designed to inhibit the AP selectively by binding to cells opsonized with antibodies and inhibiting C3 convertase (210, 211). Successful reduction of inflammation was shown *in vivo* in mouse models of experimental arthritis, ischemia/reperfusion injury and FH-deficient mice that mimick C3 glomerulopathy (210, 212, 213).

While most engineered proteins focus on inhibiting complement-mediated diseases, complement regulators can also be used in a different way. A fusion protein consisting of FH CCP 6 -7 and the Fc region of a human IgG is designed to treat bacterial infections by activating the complement system (**Figure 3C**). This protein makes clever use of the fact that pathogenic bacteria often bind to FH CCP 6-7 in order to evade the complement system (19). In addition, the Fc domain would then be able to initiate the CP by binding to C1q, which can in turn lead to C3b deposition and phagocytosis (214). This protein has been effective in enhancing complement-mediated killing *in vivo* for *Streptococcus pyogenes*, *Haemophilus influenzae* and *Neisseria meningitidis* (214–216). The increased killing of *S. pyogenes* upon FH6-7/hFC administration demonstrate that the protein can induce opsonophagocytosis, as this Gram-positive pathogen cannot be lysed by the MAC. Furthermore, FH6-7/hFc was also shown to outcompete FH for *S. pyogenes* binding, resulting in reduced complement evasion by the pathogen. This shows that the activating effect of FH6-7/hFC on the complement system is two-fold: it reduces complement evasion by outcompeting FH for binding, and it initiates more complement activation by binding to C1q (215).

Altogether, the engineered complement regulators are designed to enhance therapeutic efficacy of the regulators with two main aims. The engineered regulators are either designed to increase the regulatory power of the original protein, or they are designed to be targeted to a specific tissue and prevent systemic effects. These engineered regulators have shown promising results in a range of *in vivo* studies, although most have not been evaluated clinically to this date.

### Potentiating and Inhibiting Complement Regulation Using Antibodies

Next to the use of plasma-derived and full-length regulators and the development of engineered therapeutics, we here discuss the therapeutic use of antibodies. While those antibodies are mainly used in development of cancer therapeutics, as will be described below, we have recently developed an antibody that could be used to treat complement-mediated aHUS. The anti-FH antibodies anti-FH.07 and FH07.1 are designed to potentiate the regulatory function of FH. The antibodies bind to FH domain 18, and enhance its activity specifically on human cells. Hence, these antibodies have the potential as a therapeutic in aHUS, especially in patients that suffer from FH dysfunction. This could be an alternative to eculizumab, which has the disadvantage of inhibiting complement activity against pathogens as well. This antibody can restore FH activity in aHUS patient sera (217). In addition, we recently showed that this potentiating antibody not only potentiates wild type FH, but also four common aHUS mutated FHs *in vitro* (218).

**Figure 4:**
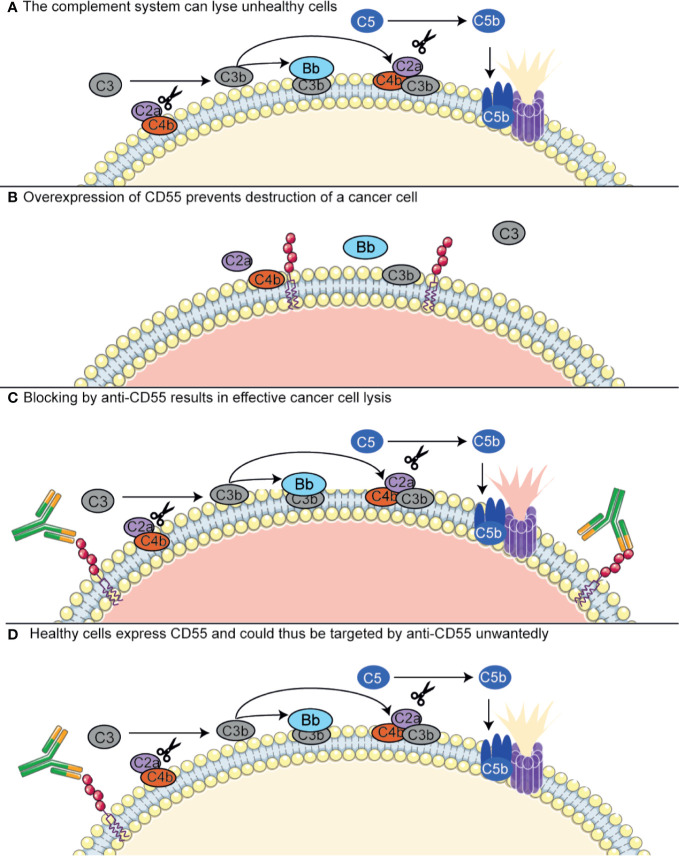
Antibodies targeting complement regulators. Antibodies targeting complement regulators can be used as immunotherapy in cancer, as some cancer types upregulate these regulators. CD55 has been shown to be overexpressed in NSCLC and colon cancer. **(A)** The complement system contributes to killing of unhealthy cells, such as cancer cells, both *via* lysis by the MAC and opsonization inducing phagocytosis (not depicted here). For MAC-mediated lysis, the complement system depends on C5 convertase (CP convertase depicted here) formation. **(B)** Cancer cells overexpressing CD55 are protected from the complement system, as CD55 has a decay accelerating activity on the C3 convertases. **(C)** An anti-CD55 antibody targets the CD55-overexpressing cancer cells and inhibits the complement regulatory activity of CD55. Thus, the cancer cells are resensitized to the complement system. **(D)** Healthy cells, conventionally expressing CD55, can also be targeted by anti-CD55 and lysed unwantedly. Figure designed using Servier Medical Art.

Most antibodies that are currently developed to target complement regulators are used to target these in cancer. As discussed above, complement regulatory proteins are often highly expressed on tumor cells and have been described to compromise the efficiency of antibody-based immunotherapies. This has raised the interests in finding blocking antibodies for the complement regulators. The effects of these antibodies can be twofold: either, complement regulators can be targeted with immunotherapy, as they are specifically upregulated in certain tumor cells, or, they can be targeted to block their function and make the tumor cells more susceptible to complement-mediated killing (**Figure 4**) (149, 154, 219, 220). However, targeting complement regulators with antibodies is not without risk. As described in the previous section, different membrane-bound regulators seem to be differentially expressed on tumor cells, but where some studies show upregulation of complement regulators, others do not. This means that usage of these antibodies would require a personalized medicine approach, in which the tumor is first extensively characterized before a drug is selected for a specific patient. More importantly, the regulators are also expressed on many other cell types throughout the body. Several studies have shown successful usage of neutralizing CD55 or CD59 antibodies in colon cancer or NSCLC, but these were investigated in cell lines or in mouse xenograft models. Here, antibodies directed against the human regulators would give rise to only very limited off-target effects, as the antibodies will only minimally recognize the mouse homologs (149, 221, 222). In addition to complement regulatory inhibiting antibodies, oligonucleotides such as anti-sense phosphorothioate oligonucleotides (S-ODNs) and small interfering RNAs (siRNAs) have been designed to silence to complement regulators. Successful silencing has been shown *in vitro*, but further developments with regards to their stability and targeting *in vivo* are still required (219, 220)

The prevention of healthy host cell targeting by these complement regulator-targeted antibodies and oligonucleotides is one of the main challenges in developing such therapeutics, as inhibiting complement regulators on healthy cells is undesirable. By administering antibodies that solely target complement regulators, a dangerous situation could arise where healthy cells expressing complement regulators are also targeted by the antibody. This poses a serious challenge for this therapeutic approach, as all healthy cells express complement regulators and most complement regulators are expressed by a majority of cells. Several possibilities are under investigation to circumvent this problem, such as bispecific antibodies or a biotin–avidin system, or circumventing the usage of antibodies altogether (220). Firstly, the bispecific antibodies are composed of Fabs of two different monoclonal antibodies: one that recognizes a complement regulator, and another that targets a tumor-specific antigen. These showed to be promising in animal models, but have not been clinically tested yet (220, 223, 224). Another possible strategy is to use a biotin–avidin system, which combines biotin-labeled mini-antibodies against CD55 and CD59 with biotin-rituximab, which targets CD20, and avidin. The mini-antibodies do not work independently, but only when combined with biotin-rituximab *via* avidin. This was shown to be effective in an *in vivo* model of B-cell lymphoma (225).

Summarizing, one potentiating antibody for a complement regulator has been successfully produced, targeting FH. The development of complement regulator inhibiting antibodies to be used as a cancer therapeutic is however still in its infancy. Several practical challenges remain to be overcome in order to test these antibodies in the clinic.

## Discussion

In conclusion, this review described the wide range of human pathologies involving complement regulators. Most of the pathologies identified affect tissues where complement or complement regulation is organized in a different way than in the rest of the body, such as the kidney, eyes and brain (28, 81, 122). The role of complement regulators in cancer was also discussed, however, there is no consensus yet on the role of complement itself in cancer (137–139) and the current evidence does not allow us to make a general statement about the role of complement regulators in cancer, which seems to differ per cancer type.

All in all, a diverse group of complement regulatory therapeutics is currently in development (4, 42, 164). There are however still challenges to overcome before these therapeutics can be used in the clinic. Although the effects of plasma-derived and recombinant molecules are highly similar, with recombinant molecules easier to produce, a shortcoming of the recombinant regulators is that their half-life is often much shorter (171, 185), which might hinder their clinical applications. In addition, the targeting or inhibiting of complement regulatory proteins in cancer is still in its infancy. Although some promising results have been shown *in vitro* and *in vivo* (149, 154, 219–221), current knowledge on expression and role of the regulators in different tumor types is lacking, which should be further clarified to develop future therapeutics. However, other drugs have found clever ways to ensure specificity of the complement regulatory effect, as has been described for the fusion proteins. The therapeutics described here have not been clinically evaluated, with the exception of recombinant soluble CR1, which would be interesting possibilities for future research (156). Their clinical success remains to be seen, as for most pathologies described in this paper targeting the complement system has not yet been proven a successful strategy. An interesting point of contention is whether this lack of success can be contributed to the specific complement modulators used in previous studies, for example in AMD, or whether a different treatment strategy should be pursued altogether.

The current research also indicates some starting points for the development of future therapeutics, if the abovementioned limitations with regards to specificity and half-time can be overcome. One clear shortcoming of the currently available therapeutics, is that there is no possibility to target the complement system at the C3 level specifically on the target tissue. As has been described above, there is a clear need for this in specific groups of PNH patients, that suffer from extravascular hemolysis under eculizumab treatment. Potentially, treatments based on complement regulatory proteins that target C3, could be a solution there, as for example FH-CR2, miniFH and anti-FH07 (197, 218). Next, the successful *in vivo* tests of FH-IgG chimeric proteins in bacterial infections, indicate that there might lie success in similar approaches using C4BP, C1-INH or Vn, which all are used by bacteria in complement evasion mechanisms (19). Lastly, future research could focus at the development of plasma-purified complement regulators other than C1-INH. Successful plasma-purification could especially be useful for FH and FI, of which mutations have been shown to affect several eye and kidney diseases and of which recombinant variants have not yet been successfully tested in a clinical setting (106, 226).

To conclude, we have described the significant role of complement regulators in a broad range of diseases. Currently, there are many therapeutics under development that use complement regulators, ranging from full-length and engineered regulators to antibodies targeting complement regulators. Altogether, the current research shows several promising directions for future clinical studies and applications of complement therapeutics based on regulators.

## Author Contributions

All authors contributed to the article and approved the submitted version.

## Funding

This work is part of the research programme Aspasia with project number 015.014.069, which is (partly) financed by the Netherlands Organisation for Scientific Research (NWO).

## Conflict of Interest

The authors declare that the research was conducted in the absence of any commercial or financial relationships that could be construed as a potential conflict of interest.
